# "Mind the gap!" Evaluation of the performance gap attributable to exception reporting and target thresholds in the new GMS contract: National database analysis

**DOI:** 10.1186/1472-6963-8-131

**Published:** 2008-06-17

**Authors:** Robert Fleetcroft, Nicholas Steel, Richard Cookson, Amanda Howe

**Affiliations:** 1 Primary Care Group, School of Medicine Health Policy and Practice, University of East Anglia Norwich, UK; 2 Department of Social Policy and Social Work, University of York, UK

## Abstract

**Background:**

The 2003 revision of the UK GMS contract rewards general practices for performance against clinical quality indicators. Practices can exempt patients from treatment, and can receive maximum payment for less than full coverage of eligible patients. This paper aims to estimate the gap between the percentage of maximum incentive gained and the percentage of patients receiving indicated care (the pay-performance gap), and to estimate how much of the gap is attributable respectively to thresholds and to exception reporting.

**Methods:**

Analysis of Quality Outcomes Framework data in the National Primary Care Database and exception reporting data from the Information Centre from 8407 practices in England in 2005 – 6. The main outcome measures were the gap between the percentage of maximum incentive gained and the percentage of patients receiving indicated care at the practice level, both for individual indicators and a combined composite score. An additional outcome was the percentage of that gap attributable respectively to exception reporting and maximum threshold targets set at less than 100%.

**Results:**

The mean pay-performance gap for the 65 aggregated clinical indicators was 13.3% (range 2.9% to 48%). 52% of this gap (6.9% of eligible patients) is attributable to thresholds being set at less than 100%, and 48% to patients being exception reported. The gap was greater than 25% in 9 indicators: beta blockers and cholesterol control in heart disease; cholesterol control in stroke; influenza immunization in asthma; blood pressure, sugar and cholesterol control in diabetes; seizures in epilepsy and treatment of hypertension.

**Conclusion:**

Threshold targets and exception reporting introduce an incentive ceiling, which substantially reduces the percentage of eligible patients that UK practices need to treat in order to receive maximum incentive payments for delivering that care. There are good clinical reasons for exception reporting, but after unsuitable patients have been exempted from treatment, there is no reason why all maximum thresholds should not be 100%, whilst retaining the current lower thresholds to provide incentives for lower performing practices.

## Background

Pay for performance (PfP) incentives were introduced to UK primary care in 1991. These were initially limited to vaccinations, immunizations and cervical cancer screening. The new GMS contract introduced in 2004 represented a major change to general practice funding, with a substantial increases in PfP for quality indicators, delivered through a quality and outcomes framework (QOF) based on the best available research evidence [1]. It is a large investment in UK primary care, with an estimated cost of £1.8 billion each year [2]. In the first year of contract implementation, these quality payments accounted for approximately 25% of a typical GP principal's pay [3]. Consequently delivery of care has changed and there is evidence that quality has increased in the contract conditions but not in other areas of quality care outside the contract [4].

Practices are rewarded for performance based on quality indicators, which initially covered 10 clinical domains and aspects of practice organisation, patient experience and enhanced services. The clinical indicators in the new GMS contract are listed in Additional file 1. More than half the incentive points are allocated to clinical performance (550 out of 1050). There has been a gross payment of £124.60 per point since 2005/6, based on a typical practice of 5500 patients with a typical prevalence of disease. Practices in England achieved higher results than expected by the NHS Employers negotiating team, gaining an average of 96% of the maximum points [3]. There is a mechanism for adjusting payment to each practice based on the prevalence of disease for each indicator, though this adjustment may not reflect the workload of a practice with a significantly different prevalence from the average [5]. At first glance the achievement of practices gaining almost all of the incentive payments available suggests close to maximum performance at targeting patients at risk: but maximum points and payment can be gained in every clinical indicator *before *all eligible patients receive indicated care.

There are two mechanisms which reduce the percentage of eligible patients that practices need to treat in order to receive maximum incentive payments. First, 'exception reporting' allows clinicians to exclude patients from the treatment indicated by a clinical indicator. Exception reporting is an important process because clinicians should not be put under pressure to prescribe inappropriately to achieve an indicator. Appropriate reasons for exception reporting have been agreed between the Department of Health and the British Medical Association and these are comprehensive (Table 1) [1]. 5.55% of patients were 'exception reported' by English practices in 2005/6 [6]. There is substantial variation between practices in exception reporting rates, with 5.4% of practices exceeding an average exception reporting rate of 10% [6].

**Table 1:** Agreed criteria for exception reporting

**A**	Patients who have been recorded as refusing to attend review who have been invited on at least three occasions during the preceding twelve months
**B**	Patients for whom it is not appropriate to review the chronic disease parameters due to particular circumstances e.g. terminal illness, extreme frailty
**C**	Patients newly diagnosed within the practice or who have recently registered with the practice, who should have measurements made within three months and delivery of clinical standards within nine months e.g. blood pressure or cholesterol measurements within target levels
**D**	Patients who are on maximum tolerated doses of medication whose levels remain sub-optimal
**E**	Patients for whom prescribing a medication is not clinically appropriate e.g. those who have an allergy, another contraindication or have experienced an adverse reaction
**F**	Where a patient has not tolerated medication
**G**	Where a patient does not agree to investigation or treatment (informed dissent), and this has been recorded in their medical records
**H**	Where the patient has a supervening condition which makes treatment of their condition inappropriate e.g. cholesterol reduction where the patient has liver disease
**I**	Where an investigative service or secondary care service is unavailable

The second mechanism which reduces the percentage of eligible patients that practices need to treat in order to receive maximum incentive payment is the presence of target thresholds set at less than 100%. For each clinical indicator there is a graduated scale of payments from a minimum to maximum threshold. A practice achieves maximum points payments at thresholds lower than 100% coverage of patients eligible for all interventions, with the lowest threshold for maximum payment being 50% (indicator CHD 10) and the highest threshold being 90% in 37 indicators (Table 2). These thresholds were described as the "maximum practically achievable level to deliver clinical effectiveness" [1]. No definition of 'practically achievable' was given, and it was not intended to be the same as 'clinically desirable'. Importantly, once the maximum threshold is reached there is no financial incentive to treat more eligible patients. Of course doctors perform for other than financial reasons, and many (though not all) practices exceed the maximum target thresholds despite there being no additional payment for doing so.

**Table 2:** Threshold targets and maximum payments

Clinical indicator	Maximum points payment	Payment stages (min-max thresholds)
CHD 2	7	25–90%
CHD 3	7	25–90%
CHD 4	4	25–90%
CHD 5	7	25–90%
CHD 6	19	25–70%
CHD 7	7	25–90%
CHD 8	16	25–60%
CHD 9	7	25–90%
CHD 10	7	25–50%
CHD 11	7	25–70%
CHD 12	7	25–85%
LVD 2	6	25–90%
LVD 3	10	25–70%
STROKE 2	2	25–80%
STROKE 3	3	25–90%
STROKE 4	2	25–70%
STROKE 5	2	25–90%
STROKE 6	5	25–70%
STROKE 7	2	25–90%
STROKE 8	5	25–60%
STROKE 9	4	25–90%
STROKE 10	2	25–85%
BP2	10	25–90%
BP3	10	25–90%
BP4	20	25–90%
BP5	56	25–70%
DM 2	3	25–90%
DM 3	3	25–90%
DM 4	5	25–90%
DM 5	3	25–90%
DM 6	16	25–50%
DM 7	11	25–85%
DM 8	5	25–90%
DM 9	3	25–90%
DM 10	3	25–90%
DM 11	3	25–90%
DM 12	17	25–55%
DM 13	3	25–90%
DM 14	3	25–90%
DM 15	3	25–70%
DM 16	3	25–90%
DM 17	6	25–60%
DM 18	3	25–85%
COPD 2	5	25–90%
COPD 3	5	25–90%
COPD 4	6	25–90%
COPD 5	6	25–90%
COPD 6	6	25–70%
COPD 7	6	25–90%
COPD 8	6	25–85%
EPIL 2	4	25–90%
EPIL 3	4	25–90%
EPIL 4	6	25–70%
THY2	6	25–90%
CANCER2	6	25–90%
MH2	23	25–90%
MH3	3	25–90%
MH4	3	25–90%
MH5	5	25–70%
ASTHMA 2	15	25–70%
ASTHMA 3	6	25–70%
ASTHMA 4	6	25–70%
ASTHMA 5	6	25–70%
ASTHMA 6	20	25–70%
ASTHMA 7	12	25–70%

An example of the way in which this payment scheme operates is indicator LVD 3, the percentage of patients with a diagnosis of CHD and left ventricular dysfunction who are currently treated with ACE inhibitors, where the minimum threshold that triggers payment is 25%. There is a sliding scale of increasing payment up to a maximum of 10 points achieved at 70% uptake of the target population. The combination of exception reporting and low targets reduce the potential health gain from an indicator, as a practice always achieves maximum incentive points in a particular indicator before all eligible patients have received treatment. This is because all maximum target thresholds are set below 100% population coverage and this target is applied after exception reported patients have been excluded.

The objectives of this paper are:

1. To estimate the gap for each indicator between the percentage of maximum incentive gained, and the percentage of patients who receive indicated care.

2. to estimate how much of the gap is attributable respectively to thresholds for maximum payment set at less than 100%, and to exception reporting.

## Methods

Thresholds for maximum payments were obtained from the 2003 GMS contract [1]. Data on points gained and reported quality performance for each clinical indicator in all English practices for the year 2005/6 were obtained from the National Primary Care Database. The contract has 76 clinical indicators. Exception reporting rates for 65 clinical indicators in all English practices for the year 2005/6 were obtained from the NHS Information Centre. Exception reporting is not applicable to the remaining 11 indicators as they refer purely to setting up disease registers that are used to define the population eligible for the 65 indicators.

The 'gap' was computed for each indicator for all patients by calculating the difference between the percentage of incentive gained and the percentage of patients who received treatment. To compute the size of the gap attributable to the threshold targets the exception reporting rate was deducted from the total gap for each indicator. The computations used to determine the numbers of patients receiving care are displayed in Table 3. Ethical approval was not needed for this study as all data was available in the public domain.

**Table 3:** computations for the pay performance gap.

For all practices {i = 1...I} and indicators {j = 1...J}:
Number of patients eligible for treatment before exceptions, N_ij_
Number of patients receiving treatment, X_ij_
Number of exceptions, E_ij_
Exception reporting rate (proportion), E_ij_/N_ij _= e_ij_
Actual quality performance score before exceptions, q_ij _= X_ij_/N_ij_
Reported quality performance score after exceptions, q^R ^_ij _= X_ij_/N_ij _- E_ij_
Rearranging gives X_ij _= q^R ^_ij _* (N_ij _- E_ij_)
Therefore actual quality performance qij=qijR∗(Nij−Eij)/Nij=qijR∗(1−Eij/Nij)=qijR∗(1−eij)
Pay score, p_ij _= (actual pay/maximum pay) for practice i and indicator j
Pay-performance gap for practice i and indicator j, g_ij _= p_ij _- q_ij_
Pay-performance gap for indicator j, g_j _= ∑_i _p_ij _- q_ij_
Pay-performance gap for practice i, g_i _= ∑_j _p_ij _- q_ij_

## Results

The pay-performance gap for each indicator, and the amount of this gap attributable to exception reporting and target thresholds respectively are given in Table 4. At the indicator level, the mean pay:performance gap for the 65 indicators was 13.3% (s.d. 9.8). The pay:performance gap was greater than 25% in nine indicators, which included indicators with interventions with significant health gain. These pay:performance gaps were respectively; 48% for beta blockers in heart disease, 43% for glucose control in diabetes, 35% for cholesterol control after a stroke, 34% for influenza immunization in asthma, 30% for blood pressure control in diabetes, 29% in seizure control in epilepsy, 29% in cholesterol control in diabetes, 29% in cholesterol control in heart disease and 26% in blood pressure control in hypertension.

**Table 4:** Mean pay: performance gap by indicator

Clinical indicator	Mean pay: performance gap %	Standard deviation	% of gap attributed to exception reporting	% of gap attributed to target thresholds <100%
CHD 2	11.7	13.6	11.4	0.3
CHD 3	4.6	3.3	1.6	3.0
CHD 4	5.4	5.7	2.7	2.7
CHD 5	3.5	2.7	1.3	2.2
CHD 6	16.2	6.7	4.1	12.1
CHD 7	8.2	4.4	3.4	4.8
CHD 8	29.0	8.6	10.2	18.8
CHD 9	8.2	3.8	3.6	4.6
CHD 10	48.0	9.0	25.2	22.8
CHD 11	17.1	11.2	7.3	9.8
CHD 12	17.6	6.0	10.7	6.9
LVD 2	10.2	15.0	10.3	-0.1
LVD 3	19.0	11.2	7.6	11.4
STROKE 2	15.1	13.6	10.8	4.3
STROKE 3	6.3	4.5	2.8	3.5
STROKE 4	9.6	10.2	3.5	6.1
STROKE 5	5.1	4.1	2.2	2.9
STROKE 6	19.9	8.6	6.7	13.2
STROKE 7	10.4	7.3	6.2	4.2
STROKE 8	34.8	11.6	14.7	20.1
STROKE 9	9.1	6.0	4.9	4.2
STROKE 10	20.1	8.1	13.4	6.7
BP2	4.3	2.8	1.6	2.7
BP3	2.9	3.0	0.7	2.2
BP4	7.3	2.8	1.0	6.3
BP5	25.7	6.8	5.5	20.2
DM 2	7.8	4.4	3.5	4.3
DM 3	4.0	3.0	1.7	2.3
DM 4	5.8	5.7	3.2	2.6
DM 5	6.3	3.9	3.3	3.0
DM 6	43.4	10.3	12.7	30.7
DM 7	13.6	5.4	6.5	7.1
DM 8	10.2	7.7	6.5	3.7
DM 9	9.6	7.3	6.3	3.3
DM 10	9.6	7.6	6.4	3.2
DM 11	3.3	2.7	1.6	1.7
DM 12	30.4	9.5	8.0	22.4
DM 13	9.8	11.4	7.6	2.2
DM 14	6.1	3.9	2.6	3.5
DM 15	13.7	12.6	5.3	8.4
DM 16	6.7	3.8	2.9	3.8
DM 17	29.0	8.1	10.6	18.4
DM 18	19.9	6.6	13.2	6.7
COPD 2	10.2	11.5	9.4	0.8
COPD 3	10.4	9.9	8.4	2.0
COPD 4	5.2	4.2	2.3	2.9
COPD 5	4.9	5.9	2.5	2.4
COPD 6	17.8	12.4	9.1	8.7
COPD 7	8.4	7.5	5.5	2.9
COPD 8	16.7	7.5	11.0	5.7
EPILEPSY 2	6.2	6.6	3.6	2.6
EPILEPSY 3	6.2	6.6	3.5	2.7
EPILEPSY 4	29.1	17.2	17.4	11.7
THYROID2	4.2	3.2	0.7	3.5
CANCER2	11.1	10.2	9.2	1.9
MH2	7.7	9.6	5.0	2.7
MH3	4.7	10.0	4.2	0.5
MH4	6.4	15.6	3.4	3.0
MH5	12.2	14.7	8.1	4.1
ASTHMA 2	11.7	8.6	3.6	8.1
ASTHMA 3	15.9	10.2	2.5	13.4
ASTHMA 4	7.6	5.0	1.3	6.3
ASTHMA 5	13.0	8.2	2.4	10.6
ASTHMA 6	21.7	9.5	4.4	17.3
ASTHMA 7	33.6	11.9	18.0	15.6

**Figure 1:**
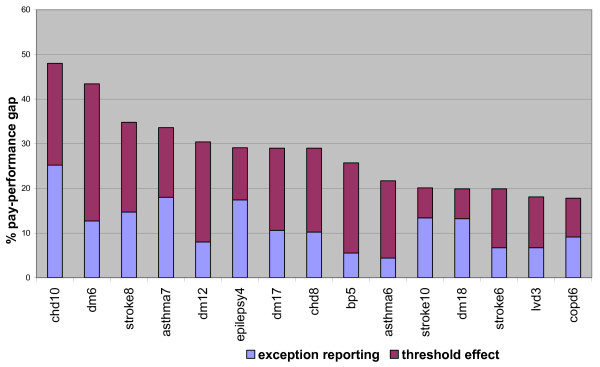
pay: performance gap for top 15 indicators.

Thresholds below 100% account for 52% of the pay:performance gap, and exception reporting accounts for the remaining 48% of the pay:performance gap. Figure 1 shows the top 15 indicators with the largest pay:performance gap and the separate contribution made by exception reporting and thresholds.

## Discussion

This is the first study of its kind to examine the gap between the percentage of financial incentive gained by each practice and the percentage of patients in that practice who receive indicated care. The strength of this study is that it uses data routinely collected from every practice in England for each clinical indicator which has exception reporting data. The mean gap between the percentage of maximum incentive that a practice receives and the percentage of eligible patients receiving care is 13.3%. This gap exceeds 25% in nine indicators. Five of these nine indicators with the largest pay-performance gap have an evidence base linking them to reductions in mortality. These five indicators are beta-blockers in heart disease (relative risk reduction 24%) [7], glucose control in diabetes (relative risk reduction 36%) [8], blood pressure control in diabetes (relative risk reduction 35%) [9], cholesterol control in heart disease (relative risk reduction 24%) [7] and blood pressure control in hypertension (relative risk reduction 16%) [19]. In all of these indicators the target threshold being set at less than 100% has contributed significantly to this gap between the financial incentive gained and number of patients receiving treatment.

The implication of these findings is that maximum target thresholds set at less than 100% may be contributing to reducing health gain for patients. Maximum target thresholds account for 52% and exception reporting accounts for 48% of the pay-performance gap respectively. It seems unlikely that such a large gap can be attributed to patients who are clinically unsuitable or unwilling to accept care for their condition as these patients should be accounted for by exception reporting. The combination of exception reporting and threshold targets set below 100%, while perhaps thought to be overambitious before implementation, appears now to be in danger of producing an incentive ceiling effect, where maximum payment is received for less than maximum coverage of the eligible population. This could reduce the health gain from the new GMS contract as we have observed both high exception reporting rates and low target thresholds in some indicators with significant intermediate health gain outcomes.

Exception reporting is patient specific and has the advantage of being sensitive to patients needs when it is used appropriately. The agreed criteria for exception reporting are wide ranging and will cover most circumstances where patients are not suitable for a particular indicator. Since primary care has achieved maximum points in many areas, there may be no rationale for maximum target thresholds to be set below 100% as there are comprehensive reasons for exception reporting any patient who would not theoretically benefit from the indicated care. In the 2006/7 revision to the GMS contract several of the maximum thresholds have been increased to 90%, though lower maximum thresholds still persist for several clinical indicators [11].

This work adds significant new information to previous published work on exception reporting and UK primary care. One study based in 1024 Scottish general practices found that when exception reporting is taken into account there was lower delivered quality of care in less affluent practices in 4 clinical indicators [12]. A further study based in one English primary care trust found higher exception reporting rates in diabetic populations with higher deprivation [13]. These 2 studies differ from ours in that we studied the effect of target thresholds on the percentage of patients receiving indicated care, as well as exception reporting. We used actual rather than estimated exception reporting data. We included a significantly larger number of practices (8407), and included all 65 clinical indicators for which exception reporting occurs, where as McClean et al included 33 indicators and Sigfrid et al included 15 indicators.

## Conclusion

The combination of both exception reporting and target thresholds set at less than 100% lead to a misalignment of incentives in the GMS contract for UK primary care, where maximum incentive payment is reached before all eligible patients have received appropriate treatment or health care intervention. There is a high exception reporting rate in several indicators with significant intermediate health outcomes which also have low maximum target thresholds. The policy implication is that all maximum target thresholds should be set at 100%, whilst retaining the current lower thresholds to provide incentives for lower performing practices. Appropriate exception reporting is likely to be unavoidable and necessary, and should be retained for patients unsuitable for a particular indicator. Incentive payments would then be provided for 100% of eligible patients in the population.

## Abbreviations

GMS: General Medical Services; PfP: pay for performance; QOF: quality and outcomes framework; UK: United Kingdom.

## Competing interests

All authors have received funding from the Department of Health for a study estimating the health impact gain of the new GMS contract

## Authors' contributions

Contributors: RF developed the study concept and design, supervised the study, obtained and analysed the data, wrote the paper and is the guarantor. NS, RC and AH advised on the study design, and the interpretation of the results. All authors contributed to, read and approved the final manuscript.

## Pre-publication history

The pre-publication history for this paper can be accessed here:



## Supplementary Material

Additional file 1 Clinical Indicators in the 2003 GMS contract. Click here for file
