# Supplementary material for: "Mind the gap!" Evaluation of the performance gap attributable to exception reporting and target thresholds in the new GMS contract: National database analysis
Source: BMC Health Serv Res. 2008 Jun 17;8:131. doi: 10.1186/1472-6963-8-131 (PMC2442837; doi:10.1186/1472-6963-8-131)
Supplement: Additional file 1 — Clinical Indicators in the 2003 GMS contract. [file 1472-6963-8-131-S1.doc]

# Additional file 1. Clinical Indicators in the 2003 GMS contract

| **clinical indicator** | **Full description** |
| --- | --- |
| CHD 1 | The practice can produce a register of patients with coronary heart disease |
| CHD 2 | The percentage of patients with newly diagnosed angina (diagnosed after 1 April 2003) who are referred for exercise testing and/or specialist assessment |
| CHD 3 | The percentage of patients with coronary heart disease whose notes record smoking status in the past 15 months, except those who have never smoked where smoking status need be recorded only once |
| CHD 4 | The percentage of patients with coronary heart disease who smoke, whose notes contain a record that smoking cessation advice or referral to a specialist service, where available, has been offered within the last 15 months |
| CHD 5 | The percentage of patients with coronary heart disease whose notes have a record of blood pressure in the previous 15 months |
| CHD 6 | The percentage of patients with coronary heart disease in whom the last blood pressure reading (measured in the last 15 months) is 150/90 or less |
| CHD 7 | The percentage of patients with coronary heart disease whose  notes have a record of total cholesterol in the previous 15 months |
| CHD 8 | The percentage of patients with coronary heart disease whose last measured total cholesterol (measured in last 15 months) is 5 mmol/l or less |
| CHD 9 | The percentage of patients with coronary heart disease with a record in the last 15 months that aspirin, an alternative anti-platelet therapy, or an anti-coagulant is being taken (unless a contraindication or side-effects are recorded) |
| CHD 10 | The percentage of patients with coronary heart disease who are currently treated with a beta blocker (unless a contraindication or side-effects are recorded) |
| CHD 11 | The percentage of patients with a history of myocardial infarction (diagnosed after 1 April 2003) who are currently treated with an ACE inhibitor |
| CHD 12 | The percentage of patients with coronary heart disease who have a record of influenza immunisation in the preceding 1 September to 31 March |
| LVD 1 | The practice can produce a register of patients with CHD and left ventricular dysfunction |
| LVD 2 | The percentage of patients with a diagnosis of CHD and left ventricular dysfunction (diagnosed after 1 April 2003) which has been confirmed by an echocardiogram |
| LVD 3 | The percentage of patients with a diagnosis of CHD and left ventricular dysfunction who are currently treated with ACE inhibitors (or A2 antagonists) |
| STROKE1 | The practice can produce a register of patients with Stroke or TIA |
| STROKE 2 | The percentage of new patients with presumptive stroke (presenting after 1 April 2003) who have been referred for confirmation of the diagnosis by CT or MRI scan |
| STROKE 3 | The percentage of patients with TIA or stroke who have a record of smoking status in the last 15 months, except those who have never smoked where smoking status should be recorded at least once since diagnosis |
| STROKE 4 | The percentage of patients with a history of TIA or stroke who smoke and whose notes contain a record that smoking cessation advice or referral to a specialist service, if available, has been offered in the last 15 months |
| STROKE 5 | The percentage of patients with TIA or stroke who have a record of blood pressure in the notes in the preceding 15 months |
| STROKE 6 | The percentage of patients with a history of TIA or stroke in whom the last blood pressure reading (measured in last 15 months) is 150/90 or less |
| STROKE 7 | The percentage of patients with TIA or stroke who have a record of total cholesterol in the last 15 months |
| STROKE 8 | The percentage of patients with TIA or stroke whose last measured total cholesterol (measured in last 15 months) is 5 mmol/l or less |
| STROKE 9 | The percentage of patients with a stroke shown to be non haemorrhagic, or a history of TIA, who have a record that aspirin, an alternative anti-platelet therapy, or an anti-coagulant is being taken (unless a contraindication or side-effects are recorded) |
| STROKE 10 | The percentage of patients with TIA or stroke who have had influenza immunisation in the preceding 1 September to 31 March |
| BP1 | The practice can produce a register of patients with established  hypertension |
| BP2 | The percentage of patients with hypertension whose notes record smoking status at least once |
| BP3 | The percentage of patients with hypertension who smoke, whose notes contain a record that smoking cessation advice or referral to a specialist service, if available, has been offered at least once |
| BP4 | The percentage of patients with hypertension in whom there is a record of the blood pressure in the past 9 months |
| BP5 | The percentage of patients with hypertension in whom the last blood pressure (measured in the last 9 months) is 150/90 or less |
| DM1 | The practice can produce a register of all patients with diabetes  mellitus |
| DM 2 | The percentage of patients with diabetes whose notes record BMI in the previous 15 months |
| DM 3 | The percentage of patients with diabetes in whom there is a record of smoking status in the previous 15 months, except those who have never smoked where smoking status should be recorded once |
| DM 4 | The percentage of patients with diabetes who smoke and whose notes contain a record that smoking cessation advice or referral to a specialist service, where available, has been offered in the last 15 months |
| DM 5 | The percentage of diabetic patients who have a record of HbA1c or equivalent in the previous 15 months |
| DM 6 | The percentage of patients with diabetes in whom the last HbA1C is 7.4 or less (or equivalent test/reference range depending on local laboratory) in last 15 months |
| DM 7 | The percentage of patients with diabetes in whom the last HbA1C is 10 or less (or equivalent test/reference range depending on local laboratory) in last 15 months |
| DM 8 | The percentage of patients with diabetes who have a record of retinal screening in the previous 15 months |
| DM 9 | The percentage of patients with diabetes with a record of the presence or absence of peripheral pulses in the previous 15 months |
| DM 10 | The percentage of patients with diabetes with a record of neuropathy testing in the previous 15 months |
| DM 11 | The percentage of patients with diabetes who have a record of the blood pressure in the past 15 months |
| DM 12 | The percentage of patients with diabetes in whom the last blood pressure is 145/85 or less |
| DM 13 | The percentage of patients with diabetes who have a record of micro-albuminuria testing in the previous 15 months (exception reporting for patients with proteinuria) |
| DM 14 | The percentage of patients with diabetes who have a record of serum creatinine testing in the previous 15 months |
| DM 15 | The percentage of patients with diabetes with proteinuria or micro-albuminuria who are treated with ACE inhibitors (or A2 antagonists) |
| DM 16 | The percentage of patients with diabetes who have a record of total cholesterol in the previous 15 months |
| DM 17 | The percentage of patients with diabetes whose last measured total cholesterol within the previous 15 months is 5mmol/l or less |
| DM 18 | The percentage of patients with diabetes who have had influenza immunisation in the preceding 1 September to 31 March |
| COPD1 | The practice can produce a register of patients with COPD |
| COPD 2 | The percentage of patients in whom diagnosis has been confirmed by spirometry including reversibility testing for newly diagnosed patients with effect from 1 April 2003 |
| COPD 3 | The percentage of all patients with COPD in whom diagnosis has been confirmed by spirometry including reversibility testing |
| COPD 4 | The percentage of patients with COPD in whom there is a record of smoking status in the previous 15 months |
| COPD 5 | The percentage of patients with COPD who smoke, whose notes contain a record that smoking cessation advice or referral to a specialist service, if available, has been offered in the past 15 months |
| COPD 6 | The percentage of patients with COPD with a record of FeV1 in the previous 27 months |
| COPD 7 | The percentage of patients with COPD receiving inhaled treatment in whom there is a record that inhaler technique has been checked in the preceding 27 months |
| COPD 8 | The percentage of patients with COPD who have had influenza immunisation in the preceding 1 September to 31 March |
| EPIL1 | The practice can produce a register of patients receiving drug treatment for epilepsy |
| EPIL 2 | The percentage of patients aged 16 and over on drug treatment for epilepsy who have a record of seizure frequency in the previous 15 months |
| EPIL 3 | The percentage of patients aged 16 and over on drug treatment for epilepsy who have a record of medication review in the previous 15 months |
| EPIL 4 | The percentage of patients aged 16 and over on drug treatment for epilepsy who have been seizure free for the last 12 months recorded in the last 15 months |
| THY1 | The practice can produce a register of patients with hypothyroidism |
| THY2 | The percentage of patients with hypothyroidism with thyroid function tests recorded in the previous 15 months |
| CANCER1 | The practice can produce a register of all cancer patients diagnosed after 1 April 2003 |
| CANCER2 | The percentage of patients with cancer diagnosed from 1 April 2003 with a review by the practice recorded within six months of confirmed diagnosis. This should include an assessment of support needs, if any, and a review of co-ordination arrangements with secondary care |
| MH1 | The practice can produce a register of people with severe long term  mental health problems who require and have agreed to regular follow-up |
| MH2 | The percentage of patients with severe long-term mental health problems with a review recorded in the preceding 15 months. This review includes a check on the accuracy of prescribed medication, a review of physical health and a review of co-ordination arrangements with secondary care |
| MH3 | The percentage of patients on lithium therapy with a record of lithium levels checked within the previous 6 months |
| MH4 | The percentage of patients on lithium therapy with a record of serum creatinine and TSH in the preceding 15 months |
| MH5 | The percentage of patients on lithium therapy with a record of lithium levels in the therapeutic range within the previous 6 months |
| ASTHMA1 | The practice can produce a register of patients with asthma, excluding patients with asthma who have been prescribed no asthma related drugs in the last twelve months |
| ASTHMA 2 | The percentage of patients aged eight and over diagnosed as having asthma from 1 April 2003 where the diagnosis has been confirmed by spirometry or peak flow measurement |
| ASTHMA 3 | The percentage of patients with asthma between the ages of 14 and 19 in whom there is a record of smoking status in the previous 15 months |
| ASTHMA 4 | The percentage of patients aged 20 and over with asthma whose notes record smoking status in the past 15 months, except those who have never smoked where smoking status should be recorded at least once |
| ASTHMA 5 | The percentage of patients with asthma who smoke, and whose notes contain a record that smoking cessation advice or referral to a specialist service, if available, has been offered within the last 15 months |
| ASTHMA 6 | The percentage of patients with asthma who have had an asthma review in the last 15 months |
| ASTHMA 7 | The percentage of patients aged 16 and over with asthma who have had influenza immunisation in the preceding 1 September to 31 March |
